# Real-time seroprevalence and exposure levels of emerging pathogens in infection-naive host populations

**DOI:** 10.1038/s41598-021-84672-1

**Published:** 2021-03-12

**Authors:** Francesco Pinotti, Uri Obolski, Paul Wikramaratna, Marta Giovanetti, Robert Paton, Paul Klenerman, Craig Thompson, Sunetra Gupta, José Lourenço

**Affiliations:** 1 Department of Zoology, University of Oxford, Oxford, UK; 2 School of Public Health, Tel Aviv University, Tel Aviv, Israel; 3 Porter School of the Environment and Earth Sciences, Tel Aviv University, Tel Aviv, Israel; 4 Laboratório de Genética Celular e Molecular, Universidade Federal de Minas Gerais, Belo Horizonte, Brazil; 5 Laboratório de Flavivírus, Instituto Oswaldo Cruz Fiocruz, Rio de Janeiro, Brazil; 6 Nuffield Department of Medicine, Peter Medawar Building for Pathogen Research, Oxford, UK

**Keywords:** Infectious diseases, Computational models, Infection

## Abstract

For endemic pathogens, seroprevalence mimics overall exposure and is minimally influenced by the time that recent infections take to seroconvert. Simulating spatially-explicit and stochastic outbreaks, we set out to explore how, for emerging pathogens, the mix of exponential growth in infection events and a constant rate for seroconversion events could lead to real-time significant differences in the total numbers of exposed versus seropositive. We find that real-time seroprevalence of an emerging pathogen can underestimate exposure depending on measurement time, epidemic doubling time, duration and natural variation in the time to seroconversion among hosts. We formalise mathematically how underestimation increases non-linearly as the host’s time to seroconversion is ever longer than the pathogen’s doubling time, and how more variable time to seroconversion among hosts results in lower underestimation. In practice, assuming that real-time seroprevalence reflects the true exposure to emerging pathogens risks overestimating measures of public health importance (e.g. infection fatality ratio) as well as the epidemic size of future waves. These results contribute to a better understanding and interpretation of real-time serological data collected during the emergence of pathogens in infection-naive host populations.

## Introduction

According to well established theoretical epidemiology concepts, a pathogen’s effective transmission potential is linked to the individual-level protection offered by previous exposure to the pathogen, and thus the current population-level exposure, or herd-immunity in the host population. Manipulating population level herd-immunity against pathogens, e.g. via vaccination, is a major cornerstone of contemporary epidemiology and public health efforts towards sustainable control and eradication. While the relationships of exposure with immunity (protection), and exposure with seropositivity are not necessarily straight-forward and are pathogen-specific, seroepidemiology to monitor the exposure level to particular pathogens is commonly applied in parallel to clinical and laboratory-based surveillance of new cases^1^.

During pandemics, real-time discovery, monitoring and control of transmission events is imperative. Case detection (clinical, laboratory) provides opportunities for real-time contact tracing and quarantine efforts towards elimination of transmission clusters^2–7^, but it can critically suffer from biases that arise from variation in health-seeking behaviours, health system infrastructure and capacity, symptomatic rates of infection, etc. While valuable insights such as the case fatality ratio can be obtained from case data, serology can ultimately resolve uncertainty around the true number of past infections. It can thus help define better denominators for other measures of public health importance, such as the infection fatality ratio and transmission-related parameters that serve as input to epidemiological models that guide decision-making (e.g. R0, Re). Examples from recent pandemics include the use of serology for estimation of H1N1pdm age-specific infection leading to adequate denominators for fatality, risk and symptomatic rates^8^, the revision from high to low ZIKV-associated risk of microcephaly once serology confirmed extremely high levels of local exposure to the virus (e.g.^9^, also predicted by modelling^10,11^), and the search for neutralizing antibodies (and protection) against SARS-CoV-2 in particular clinical or population sub-groups^12,13^.

By providing means of assessing the current level of exposure to an emerging pathogen, serology also informs on the likelihood of secondary epidemic waves, and can be used to quantify the (cost-)effectiveness of reducing transmission with non-pharmaceutical interventions such as personal protective equipment usage, social distancing, school closure, travel bans and more generally, societal lockdown. In practice, serology carries a number of challenges which are particularly acute for emerging pathogens^1,14–16^. For instance, capacity for serological testing is unlikely to be developed or optimised soon after pathogen emergence, with early methods more likely to suffer from specificity and sensitivity issues leading to both under and overestimation of exposure—a major concern in the ongoing SARS-CoV-2 pandemic^16^. Early in emerging epidemics, targeted cohorts for serological surveys are also less likely to be representative of the overall population, as limits in capacity and infrastructure or clinical properties of the new pathogen may dictate priority testing for particular groups in society. An assumption of zero seroprevalence (and of no immunity) before emergence of the novel pathogen may also be problematic, since many pathogens with pandemic potential are genetically related to endemic human pathogens—a fact evidenced by some of the large epidemics of this century, e.g. ZIKV, H1N1pdm, SARS-CoV, MERS-CoV, and SARS-CoV-2.

Perhaps due to its minor role in the relationship between exposure and seroprevalence for endemic pathogens, a major challenge for interpretation of serological data that is rarely discussed in the context of emerging pathogens is time to seroconversion. For endemic pathogens with a long history of local circulation, the number of recently infected individuals is typically orders of magnitude smaller than the cumulative number of exposed. As such, seroprevalence mimics overall exposure and is minimally influenced by the time that recent infections will take to seroconvert. In contrast, for newly emerging pathogens, a mix of exponential growth in infection events with a constant rate for seroconversion events could lead to differences in the total numbers of exposed versus seropositive.

In this study we use a mathematical model of transmission to explore the theoretical, real-time relationship of exposure and seroprevalence during the emergence of a novel pathogen into an infection-naive host population. We describe and formalize how epidemic growth and time to seroconversion, independently of other epidemiological parameters, can be used to summarize the expected level of underestimation of exposure from real-time seroprevalence data alone.

## Materials and methods

### Epidemiological framework

We developed an individual-based model that follows an susceptible-exposed-infectious-recovered (SLIR) epidemiological framework for the transmission of a directly transmitted pathogen. Individuals are born susceptible (S), can acquire infection experiencing an incubation period (L) followed by an infectious period (I), after which recovery from infection gives life-long immunity (R). Seroconversion is modelled separately from transmission dynamics, with time to seroconversion (T2S) after infection assumed to follow a Gamma distribution with mean $\Gamma m$ and shape $\Gamma s$ (with $\Gamma s\to 1$ resulting in exponential-like distributions and $\Gamma s\to \infty$ resulting in bell-shaped distributions with small variance). We assume that the emergent pathogen is not (sero) cross-reactive with endemic pathogens, and that there has been no exposure in the population before the simulated introduction (i.e. seropositivity is zero before pathogen emergence). Mortality is age-dependent and population size is kept constant with each death being replaced by a newborn. The framework is stochastic, the time unit is set to one day. A spatial dimension is modelled using a 2D meta-population of $nC={C}^{2}$ (host communities). The host population is divided equally into each community *C*_*i*_ *. Individuals of a community C*_*i*_ are assumed to mix homogeneously within C_i_ and with individuals in neighbouring communities (left, right, up and down of *C*_*i*_). Thus, between community transmission is modelled only locally. The force of infection to individuals of community *C*_*i*_ *is λ*_*i*_ $= \beta ({I}_{i}+{I}_{j})/({N}_{i}+{N}_{j})$ *where β is the transmission rate,* *I*_*i*_ *is the number of infectious individuals in community i*, I_j_ the sum of infectious individuals in the neighbouring communities, *N*_*i*_ *the population size of community i*, and N_j_ the sum of the population sizes of the neighbouring communities. Parameters are summarised in Table 1 and further framework details on time to seroconversion and age-dependent mortality can be found in Supplementary File 1.

**Table 1:** Model parameters.

	Meaning	Range	Default
nC	Population structure (nC^2^)	1, 100, 144, 289, 400, 900	1 (homogeneous)
R0	Basic reproduction number	2.0, 2.5, 3.0, 3.5, 4.0	2.0
$\Gamma s$	Shape of time to seroconversion distribution	1, 3, 6, 10, 100	1 (exponential-like)
$\Gamma m$	Mean of time to seroconversion distribution	1, 7, 14, 21, 28, 35, 42	14 (days)
$\sigma$	1/$\sigma$ = infectious period	0.2, 0.3, 0.4, 0.5, 0.6	0.2 (5 days)
$\delta$	1/$\delta$ = incubation period	0.2, 0.3, 0.4, 0.5, 0.6	0.3 (3 days)
$\beta$	Transmission rate, obtained from $\beta ={R0}(\delta +\mu )(\sigma +\mu )/\delta$	–	–
$1/\mu$	Average-life span (see Supplementary File 1 for details)	–	64 years
N	Total population size	1 M	1 M (individuals)

### Measuring exposure underestimation from seroprevalence

In model simulations, the time varying cumulative number of individuals that have already been exposed ($expo(t)=L+I+R$, or $expo(t)=1-S$) and the number of individuals seroconverted ($sero(t)$) are known. As detailed below, depending on the stage of the simulated epidemic and model parameters, the number of seroconverted underestimates the number of exposed. We measure the underestimation of exposure by current seroprevalence in two ways: (1) the ratio between the exposed and seroconverted (relative exposure underestimation: REU) $REU(t)=expo(t) / sero(t)$ (expression 1), and (2) the difference between the percent of exposed and percent seroconverted (absolute exposure underestimation: AEU) $AEU(t)=(100/N)[expo(t)-sero(t)]$ (expression 2), or $AEU(t)=(100/N)sero(t)[REU(t)-1]$.

Let $\Gamma (\tau )$ be the host's distribution of time to seroconversion (T2S) after infection. Then, the cumulative seroconverted $sero(t)$ can be expressed as the convolution of T2S $\Gamma (\tau )$ and the cumulative exposed $expo(t)$, formalized as $sero(t) = {\int }_{0}^{ t}\Gamma (\tau ) expo(t-\tau ) d\tau$. During the period of exponential growth of the outbreak $expo(t)=expo(0){e}^{rt}$ with growth rate $r$, such that $sero(t) = expo(0) {\int }_{0}^{ t}\Gamma (\tau ) {e}^{(t-\tau )} d\tau$. As detailed above, we define $REU(t)$ as the ratio between the cumulative exposed $expo(t)$ and seroconverted $sero(t)$, and thus $REU(t)=1/{\int }_{0}^{ t}\Gamma (\tau ) {e}^{-r\tau } d\tau$.

From here, we used the moment generating function of $\Gamma (\tau )$, to obtain the (mean) predictive REU expression $pREU=1/(r (\Gamma m/\Gamma s) + 1{)}^{-\Gamma s}$ (expression 3) valid for the exponential phase of an outbreak. The predictive REU expression can also be expressed dependent on the ratio between epidemic doubling time ($DT$) and mean time to seroconversion $\Gamma m$, as $pREU=1/(a/(\phi \Gamma s) + 1{)}^{-\Gamma s}$ (expression 4), with $DT=a/r$, $\phi =DT/\Gamma m$ and $a=ln(2)$.

### Estimation of exponential growth from simulated epidemics

A phenomenological model of exponential growth $c(t)={C}_{0}{e}^{rt}$ (expression 5) was used to estimate the growth rate from simulated outbreaks, where $c(t)$ is cumulative incidence, $r$ the growth rate, $t$ time, and ${C}_{0}$ the initial number in the cumulative series. The growth $r$ was obtained via maximum likelihood estimation using the *optim* function in R (default parameters and method set to BFGS) and a negative log-likelihood of the data given the model defined using a negative-binomial distribution.

## Results

### Seroepidemiological dynamics and exposure underestimation

**Figure 1:**
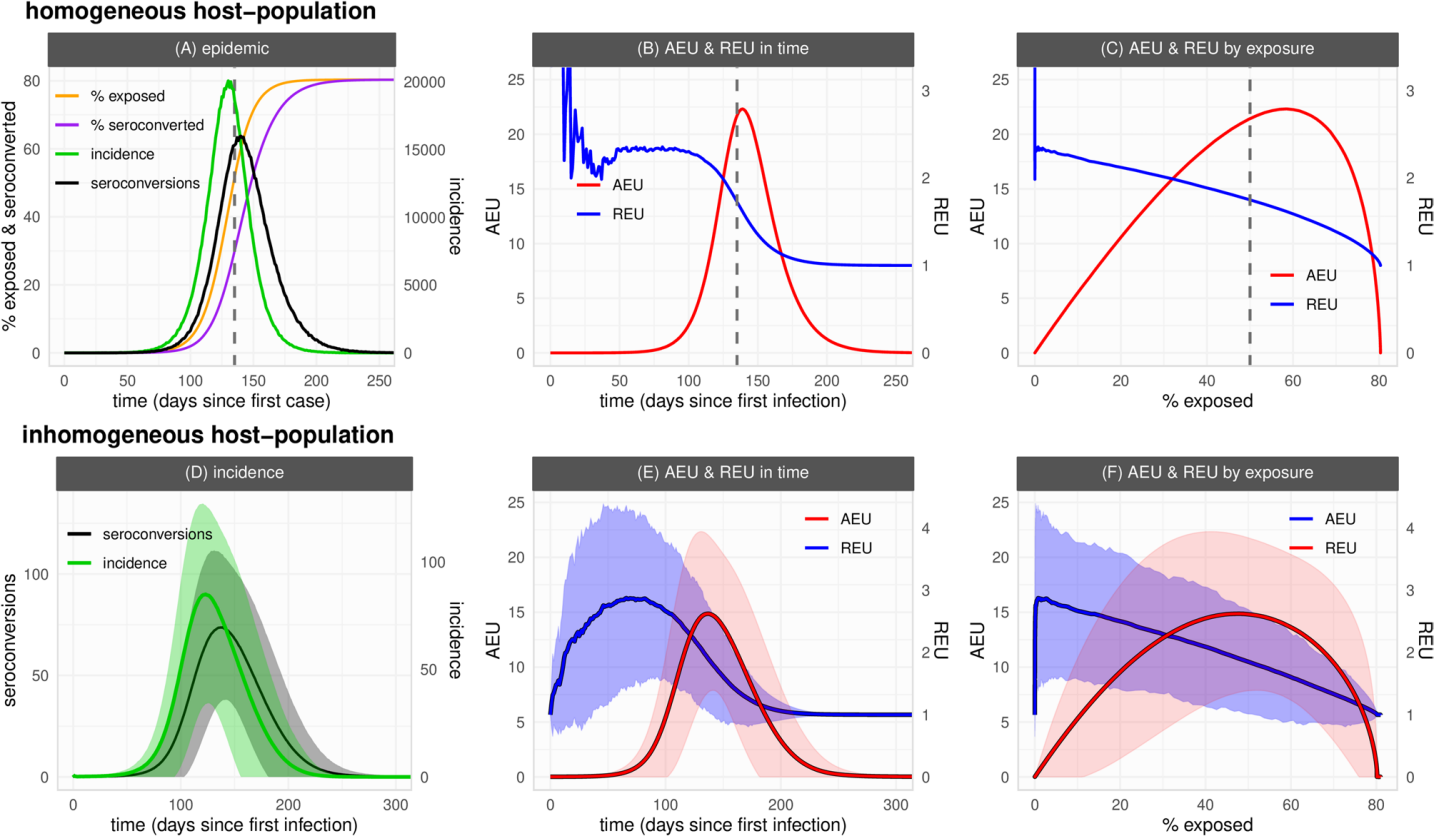
General seroepidemiological dynamics in both an homogeneous and inhomogeneous host-population. (**A**) Time series of cumulative incidence (exposed, orange), cumulative seroconverted (purple), incidence (green) and seroconversion (black) daily events. (**B**, **C**) Relative exposure underestimation (REU, blue) and absolute exposure underestimation (AEU, red) dependent on time (**B**) and the percent exposed (**C**). Outbreak simulated with parameters as default in Table 1. (**D**) Time series of incidence (green) and seroconversion (black) daily events. (**E**, **F**) REU (blue) and AEU (red) dependent on time (**E**) and the percent exposed (F). (**A**–**C**) Vertical dashed line marks the day in which the theoretical herd-immunity threshold is reached. Curves present the output of a single stochastic simulation. (**D**–**F**) Lines are the mean and areas the standard deviation of model output across all communities of the meta-population among 100 outbreaks simulated with parameters as default in Table 1 except $nC=144$ ($1{2}^{2}$ lattice).

We start by describing our framework’s seroepidemiological dynamics for a first epidemic wave of a novel emerging pathogen in an homogeneously mixing host-population (Fig. 1A–C, default parameter set as in Table 1). As expected from classic SLIR theory, incidence evolves into a bell-shaped curve that peaks at the theoretical herd-immunity threshold 1–1/R0. Up to the outbreak’s peak, both the number of daily incidence and seroconversion events increase (Fig. 1A). However, as the daily incidence starts to grow exponentially, seroconversion as dictated by a constant rate defined by the distribution of time to seroconversion (T2S) grows slower. Differences between the percent of the population already exposed and the percent already seroconverted (seroprevalence) thus accumulate in time. Later in the epidemic the exposed and seroconverted converge to the same percent as incidence events slow down and seroconversion events catch up. As such, depending on the outbreak's stage, seroprevalence may not represent a population’s current level of exposure of a novel emerging pathogen.

The level of underestimation of exposure by seroprevalence in this first epidemic wave is summarised in Figs. 1B,C, when measured by the difference between the percent of exposed and seroconverted (AEU, absolute exposure underestimation), and by the ratio between the exposed and the seroconverted (REU, relative exposure underestimation). In absolute terms (AEU), underestimation follows epidemic progression, increasing monotonically up to just after the theoretical herd-immunity threshold is reached, and declining thereafter (Fig. 1B). In relative terms (REU), underestimation can be initially erratic due to stochasticity and low number of events, but is maintained well above one during the period of epidemic growth, later converging to one (i.e. no underestimation) as the outbreak slows down after the theoretical herd-immunity threshold (Fig. 1C).

In this stochastic framework each simulation is expected to present variation in outbreak dynamics. When considering the spatial dimension, communities in the meta-population will also present variation. We thus looked at relative and absolute underestimation of exposure by seroprevalence when considering many independent runs of the framework within an inhomogeneous population (Fig. 1D–F). The variation in incidence and seroconversion events among the independent runs and communities (Fig. 1D) resulted in considerable variation in REU and AEU (Figs. 1E,F), with mean dynamic output similar to that described for the homogeneous scenario (Figs. 1A–C). As described above, either type of underestimation had its maximum during different stages of the epidemic, with REU peaking earlier during growth and AEU later near the peak.

### Summarizing exposure underestimation during epidemic growth

The results of Fig. 1 offer insights into how real-time seroprevalence during the first epidemic wave of a novel emerging pathogen can lead to significant underestimation of exposure. To characterise underestimation during epidemic growth, we introduce the predictive REU (pREU) summary measure (expressions 3–4). pREU predicts the mean underestimation of exposure by seroprevalence during the epidemic growth phase of an emerging pathogen into an infection-naive host population, given priors on an outbreak’s growth rate and the distribution of time to seroconversion (T2S).

In a sensitivity exercise, we simulated a multitude of outbreaks by varying the framework’s parameters, and for each outbreak we compared pREU with the real outbreak REU. Keeping parameters otherwise set to default (Table 1), we considered N = 100 outbreaks for each unique value of the parameters nC (meta-population structure), R0, $\Gamma m$ (T2S mean) and $\Gamma s$ (T2S shape) (as in the *Range* column of Table 1 giving a total of N = 3100 outbreaks). The growth rate $r$ of each outbreak was obtained by fitting the phenomenological model (expression 5) to the cumulative number of exposed between the time of first incidence event and outbreak peak. pREU was calculated from expression 3 using $r$, $\Gamma m$ and $\Gamma s$.

**Figure 2:**
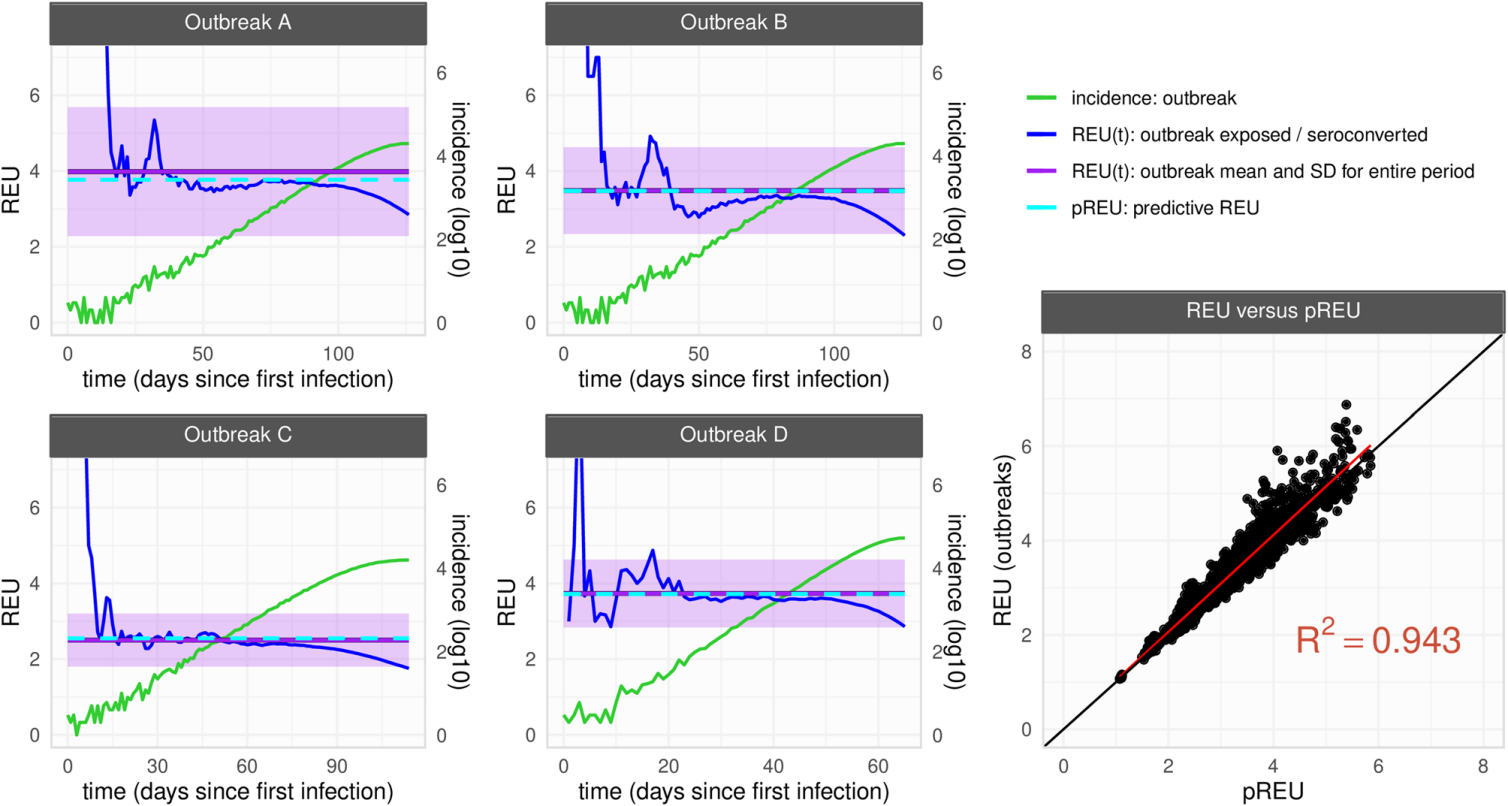
Summarizing exposure underestimation during epidemic growth. (left) Examples of simulated outbreaks (**A**–**D**) with parameter variations from the default parameter set (Fig. 1A–C, Table 1). Outbreak A with $\Gamma m=28$. Outbreak B with $\Gamma s=6$. Outbreak C with nC = 100. Outbreak D with R0 = 3.5. For each, the outbreak’s REU(t) (blue), incidence (green), REU mean and standard deviation for the entire time period (purple) and pREU (cyan) are presented. Time period considered is from the first case to outbreak peak. (right) Mean REU of each outbreak (N = 3100) and pREU shown with a linear regression with R-squared = 0.943.

All parameters had an impact on an outbreak’s REU (for the independent effects of each parameter see Figs. S3–7). In Fig. 2, four outbreak examples are presented, for which pREU is seen to approximate an outbreak’s mean REU between the time of first infection and outbreak peak. When comparing all pREU and REU from the N = 3100 outbreaks, pREU could explain ~ 94% of REU’s variation among the outbreaks generated with different host–pathogen characteristics (for the independent effects of each parameter see Figs. S8–11).

Owing to a multitude of factors (e.g. public health system, existing technology), capacity for serology testing and thus quantification of seroprevalence is likely to be better established during a second epidemic wave of an emerging pathogen, if not later. The introduced pREU summary measure applies to periods of epidemic growth independently of whether these are in first or subsequent outbreaks. It is thus able to predict the level of underestimation of exposure by seroprevalence during the growth phase of a second epidemic wave, whether it is larger (Fig. S12) or smaller (Fig. S13) than the first epidemic wave.

### Generalizing exposure underestimation during epidemic growth

Novel emerging pathogens can present significant differences in a number of epidemiological factors, such as the infectious period, transmission potential or even the mode of transmission (e.g. ZIKV versus SARS-CoV-2). Since pREU depends solely on the T2S distribution and observed epidemic growth, it allows to characterise a range of possible theoretical scenarios for an epidemic wave of a novel emerging pathogen independently of details about the aforementioned epidemiological factors. By varying the doubling time and the T2S distribution, we thus characterised pREU’s sensitivity (expression 4) to a variety of possible outbreaks by any emerging pathogen.

**Figure 3:**
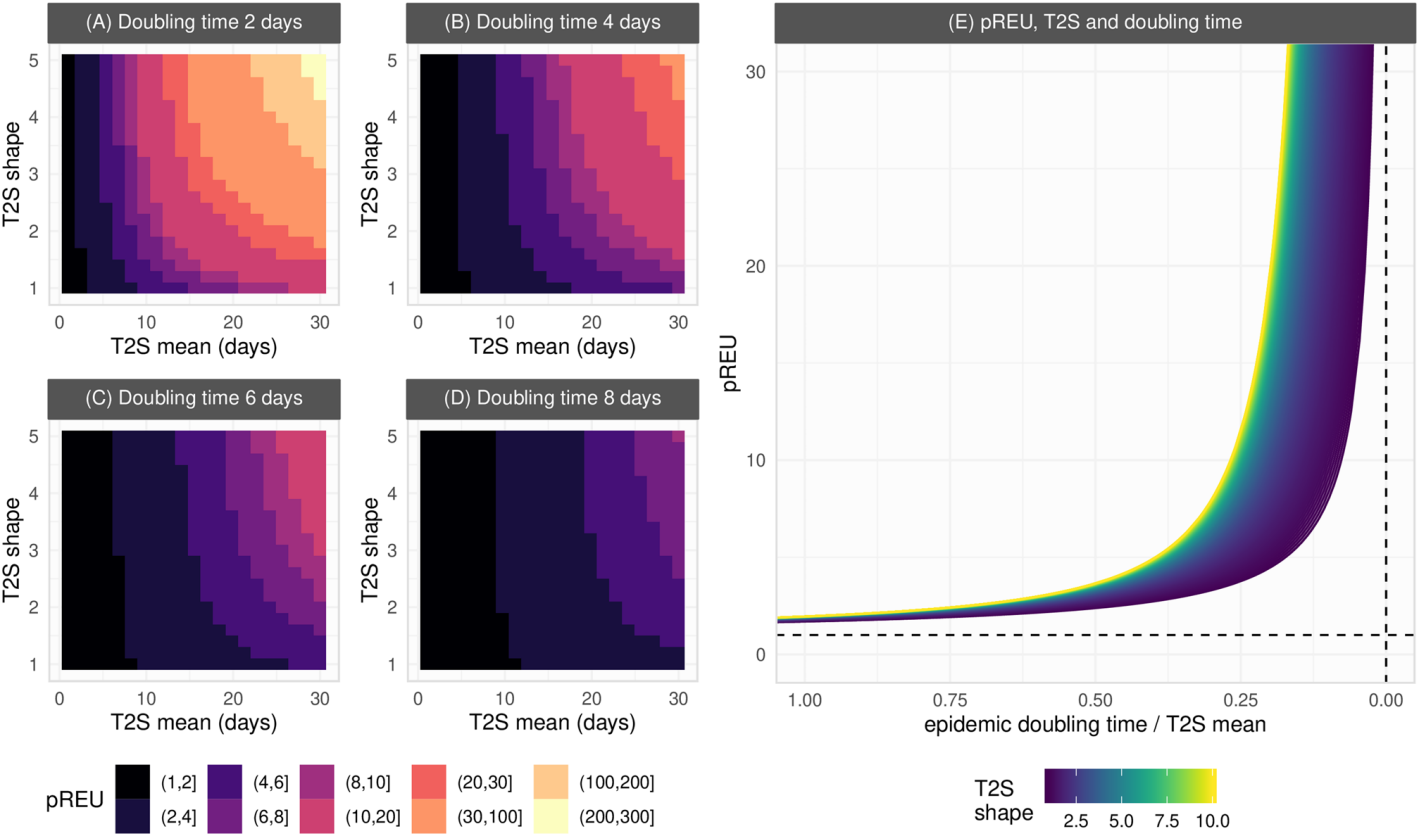
Generalizing exposure underestimation during epidemic growth. (**A**–**D**) Each heatmap of pREU is for an epidemic doubling time (panel title) and combinations of T2S mean and shape (smaller shapes imply larger variance in responses among hosts). The color scale is discretized for visualization. (**E**) Sensitivity of pREU when varying doubling time, T2S mean and shape. Dashed lines present the limits pREU = 1 and doubling time / T2S mean = 0. T2S mean varied from 1 to 100, T2S shape from 1 to 10, doubling time 1 to 100.

As summarised in Fig. 3A–D (full output in Fig. S14), pREU is the smallest for shorter times to seroconversion (smaller T2S mean). In contrast, the largest pREU is expected for combinations of longer (larger T2S mean) and less variable (larger T2S shape) time to seroconversion. As variation in seroconversion times among hosts is increased (decreasing T2S shape), pREU becomes less sensitive to the T2S mean (an effect driven by a larger proportion of hosts seroconverting earlier than the mean of the population). Overall, shorter epidemic doubling times (Fig. 3A) increase the sensitivity of pREU to both the T2S mean and shape, favouring higher underestimation of exposure by seroprevalence.

pREU’s sensitivity to properties of seroconversion time and epidemic doubling time are further illustrated in Fig. 3E when summarizing the relative duration of the two time variables. pREU is smaller when the epidemic doubling time is close to the seroconversion time, but it can quickly become large when seroconversion time is ever longer than the doubling time. The effect of seroconversion time distributions with more variance (smaller T2S shape) in reducing pREU is also seen to be significant, but this effect can be lost as seroconversion time becomes longer than the epidemic doubling time.

## Discussion

Until recently, seroepidemiology has mainly focused on endemic pathogens with a long history of circulation. Practical uses of serology have varied^1^, from informing on immunity thresholds for elimination and monitoring importation after eradication (e.g. Polio), to comparing country-level burdens of disease for international cooperation or helping draft vaccination strategies. With the recent occurrence of large epidemics and pandemics (e.g. H1N1pdm, ZIKV, SARS-CoV, SARS-CoV-2), serology has also been established to sentinel pathogens with future pandemic potential^17,18^, to quantify post-pandemic attack rates^9,19^, or to estimate epidemiological variables of interest for decision-making during epidemics^8,20^. In these scenarios characterised by the need for real-time output, seroepidemiology has faced novel challenges, including capacity and test accuracy requirements. A less explored but equally relevant challenge for real-time seroepidemiology of emerging pathogens is the time to seroconversion, which we have here explored in a theoretical framework.

We looked at underestimation of exposure by seroprevalence based on measures of both relative (REU) and absolute (AEU) difference between exposure and seroprevalence in simulated outbreaks of a novel pathogen spreading in infection-naive populations. These types of underestimation provide complementary insights into the real-time seroepidemiology of an emerging pathogen. REU demonstrates that exposure during epidemic growth can be larger than that measured by seroprevalence by a factor *X*, such that epidemiological measures that require denominators based on total number of infectious could be overestimated by the same factor *X* when assuming that real-time seroprevalence represents true exposure. Examples of such measures that are critical for capacity and response during pandemics are the infection fatality ratio or the infection hospitalization ratio^21^. In contrast, AEU demonstrates that the largest absolute errors can be made when estimating exposure from real-time seroprevalence at epidemic peaks, whether the latter are generated naturally by reaching the herd-immunity threshold, or by control initiatives suppressing transmission. Such absolute errors can be impactful on decision-making dependent on extrapolation of the level of herd-immunity achieved during the first epidemic wave. For example, modelling approaches informed by real-time seroprevalence measures near the observed first epidemic peak, risk overestimating the potential of secondary epidemic waves by assuming that seroprevalence reflects the current levels of exposure (i.e. herd-immunity).

Restricting study time to the period of epidemic growth, we were able to formalize how the combination of time to seroconversion (T2S) and epidemic growth independently of other epidemiological factors, can predict mean REU. This formulation allowed us to explore the sensitivity of real-time underestimation of exposure by seroprevalence in a generalised form, applicable to any epidemic wave of a theoretical emerging pathogen. We show that REU is expected to be ever smaller the more similar T2S is to epidemic doubling time, but critically increasing non-linearly as T2S increases relative to the doubling time. REU is also highly sensitive to variations in T2S mean and variance for fast growing outbreaks. As such, newly emerging pathogens that are characterised by short generation times and high transmissibility can have exposure significantly underestimated by real-time seroprevalence. When the variance of T2S among hosts is large, REU is generally lower and less sensitive to mean T2S, a result of large proportions of exposed hosts seroconverting much earlier than the T2S mean. Emerging pathogens characterised by large T2S variation among hosts will thus, generally, suffer from less underestimation of exposure by real-time seroprevalence.

For emerging pathogens, serology has the critical potential of validating and correcting estimates obtained by other methods on ongoing attack rates, symptomatic rates and accumulated herd-immunity within particular groups or at the population-level. Our generalised modelling exercises demonstrate how, mechanistically, real-time seroprevalence may underestimate real exposure levels depending on the timing of the measurements, the epidemic growth of the pathogen, and the distribution of time to seroconversion among hosts. These theoretical insights contribute to a better understanding and interpretation of real-time serological data collected during the emergence of pandemic pathogens.

## Limitations and future work

In this study we make a number of simplifying assumptions over the modelling framework. For example, we have used exponentially distributed periods for infection and incubation, that although are common practice in modelling exercises, may violate the real distributions of some emerging pathogens. We have also modelled a purely theoretical host-population structure (2D von Neumann lattice) akin to those used in classic meta-population studies. With it, we were able to show that while significant differences in population structure may affect epidemic progression, our main conclusions on real-time seroprevalence still hold. Host turnover (birth, deaths) has been included in the modelling framework for completion, but we note here that given the time scale analysed in our results ($<2$ years), its effect on the main conclusions is negligible. In some of the sensitivity exercises we simulate temporary reductions in the transmission rate of the emerging pathogen. These were performed with the aim of demonstrating that underestimation of exposure by seroprevalence can be present in subsequent epidemic waves, and not with the intention of demonstrating the effects of regional non-pharmaceutical interventions such as the ones implemented during the SARS-CoV-2 pandemic. We further assume that transmission and incubation is uniform across all hosts, but it is also possible that emerging pathogens may present variation among hosts, e.g. across ages, as is the case of SARS-CoV-2. Finally, we explore scenarios characterised by the emergence of a pathogen for which exposure equates to both immunity (protection) and detectable seropositivity. In reality, an emerging pathogen may face a non-zero herd-immunity (protection) landscape, may not induce life-long protection, and may (sero) cross-react with endemic pathogens. Under such alternative scenarios, there should be added uncertainty on how real-time seroprevalence may reflect real exposure levels.

Given the mathematical complexity of the problem explored, a final limitation of the current study is related to not offering a mathematical formulation for direct quantification of both the relative (REU) and absolute (AEU) difference between exposure and seroprevalence of an emerging pathogen. The general conclusion, presented over sensitivity analyses of the mathematical formulation of predictive REU (pREU) during epidemic growth, is amenable to be trivial to modellers of infectious disease transmission. Nonetheless, this study is unique in that it offers a demonstration on how, mechanistically, the underlying processes of transmission and seroconversion dictate an expected underestimation of exposure by real-time seroprevalence. This last point should be of particular value to non-modellers with interest in seroepidemiology and its relevance for real-time public health policy of emerging pathogens.

pREU remains nonetheless as a useful public health tool to estimate the ongoing underestimation of exposure by seroprevalence in future outbreaks. This is particularly the case for pathogens that, although endemic in some regions of the world, occasionally get introduced somewhere else. For such pathogens, means of testing for seropositivity may already exist before the occurrence of first epidemic waves in new locations, infection may provide life-long immunity, and (sero) cross-reactivity to endemic pathogens may be negligible. One example of the past decade includes the dengue virus serotype 1 (DENV1) outbreak in the Island of Madeira (Portugal, 2012). If seroepidemiology studies would have been performed during the emergence of the virus, pREU could have been used to correct real-time seroprevalence into real exposure levels. Together with DENV, other candidate mosquito-borne pathogens for which pREU may be useful for seroepidemiology include the Chikungunya virus (CHIKV) and ZIKV, for which ongoing climate and globalisation trends predict that future introductions into new locations will become ever more frequent.

Within the controlled setting defined by the simplifying assumptions and limitations described above, we were able to generalise the dynamics of real-time seroepidemiology for a theoretical pathogen. In general, our results are a subset of possible realities of the levels of exposure underestimation that may arise from real-time seroprevalence, and should therefore be interpreted in the context of the controlled setting they are obtained from.

## Supplementary Information

Supplementary Information

## Data Availability

There is no data on this article.
