# Supplementary material for: Real-time seroprevalence and exposure levels of emerging pathogens in infection-naive host populations
Source: Sci Rep. 2021 Mar 12;11:5825. doi: 10.1038/s41598-021-84672-1 (PMC7954847; doi:10.1038/s41598-021-84672-1)
Supplement: Supplementary file 1 — Supplementary Information [file 41598_2021_84672_MOESM1_ESM.docx]

**Real-time seroprevalence and exposure levels of emerging pathogens in infection-naive host populations**

Francesco Pinotti^#,a^, Uri Obolski^b,c^, Paul Wikramaratna*^$^*, Marta Giovanetti^d,e^, Robert Paton^a^, Paul Klenerman^f^, Craig Thompson^a^, Sunetra Gupta^a^, José Lourenço^*,#,a^

*^a^* *Department of Zoology, University of Oxford, Oxford, United Kingdom; ^b^ School of Public Health, Tel Aviv University, Tel Aviv, Israel; ^c^ Porter School of the Environment and Earth Sciences, Tel Aviv University, Tel Aviv, Israel; ^d^ Laboratório de Genética Celular e Molecular, Universidade Federal de Minas Gerais, Belo Horizonte, Brazil; ^e^ Laboratório de Flavivírus, Instituto Oswaldo Cruz Fiocruz, Rio de Janeiro, Brazil; ^f^  Nuffield Department of Medicine, Peter Medawar Building for Pathogen Research, Oxford, United Kingdom; ^$^ No affiliation.*

## **Supporting File 1:** Extra model details and analyses

### **Details on model mortality**

Mortality is age-dependent, and we use the Weibull distribution’s CDF with parameters scale=130 and shape=4.1 to model death events, leading to an average-life span of 64 years.

| 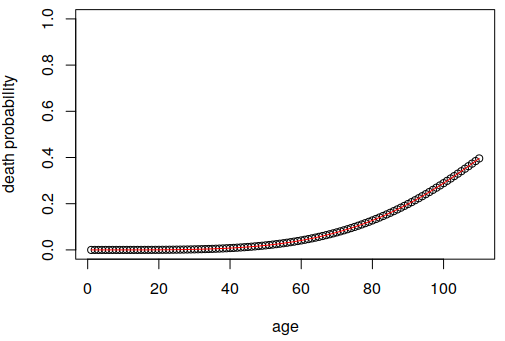 | 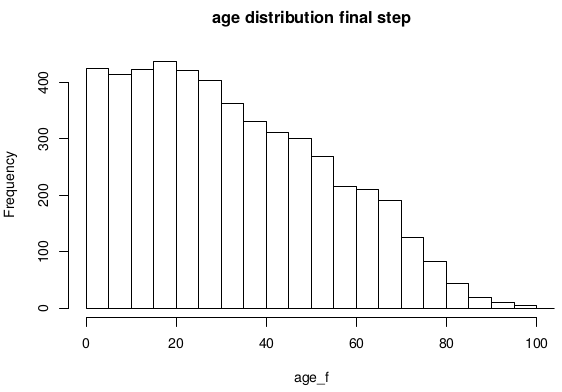 |
| --- | --- |
| Age-dependent death probability | Resulting age distribution |

**Figure S1. Example of the model’s age-dependent mortality.**

### **Details on to seroconversion time**

Time to seroconversion (T2S) is assumed to follow a Gamma distribution with mean $\Gamma m$ and shape $\Gamma s$. The choice of distribution is purely due to how easy it is to fix the mean $\Gamma m$ and change its shape from exponential-like to bell-shaped with little variance using solely its $\Gamma s$ parameter. For each simulation, incidence events are recorded and then analysed post-simulation in R assuming different T2S distributions (T2S does not affect transmission or any other mechanistic event in the model framework). General examples of used distributions with $\Gamma m=$ 14 days:

| 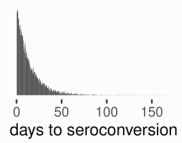  $\Gamma s=1$ | 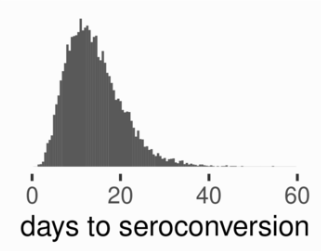$\Gamma s=5$ | 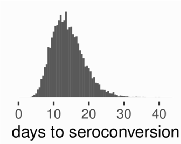$\Gamma s=10$ | 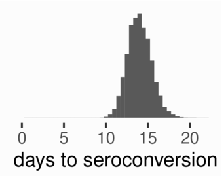$\Gamma s=100$ |
| --- | --- | --- | --- |

Figure S2. Examples of different distributions for seroconversion time.

### **Sensitivity of AEU and REU to model parameters**

Using the default parameter set (**Table 1**), we varied parameters to explore their independent effect on how seroprevalence may underestimate the true level of exposure (measuring the relative and absolute exposure underestimations - AEU, REU - like in the main text. As described in the Methods section, the assumed T2S distribution does not affect model dynamics. Thus, when varying the mean of T2S distribution ($\Gamma m$), we found no change in model epidemic output (**Figure S3A**). Both AEU and REU had a positive relationship with mean time to seroconversion, with longer times to seroconversion resulting in larger underestimations at any point of epidemic progression (percent exposed) (**Figure S3B-C**).

| 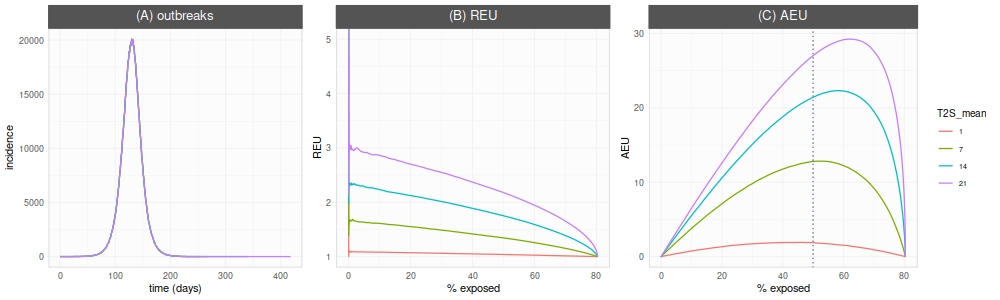  **Figure S3.** (A) Incidence time series. (B) REU = relative exposure underestimation (ratio %exposed/%seroconverted). (C) AEU = absolute exposure underestimation (%exposed - %seroconverted). Vertical dashed line is the theoretical herd-immunity threshold. (Legend) colours are the variable that is varied while all other variables are kept constant at default as in Table 1. |
| --- |

When varying the shape of T2S distribution ($\Gamma s$), as expected, we also found no change in model epidemic output (**Figure S4A**). As T2S changed from a bell-shaped distribution with low variance ($\Gamma s=100$) to exponential-like with large variance ($\Gamma s=1$), a larger proportion of incidence events seroconverted much earlier than the mean time to seroconversion. As a result, both AEU and REU had a general negative relationship with $\Gamma s$, with lower underestimations for T2S distributions with larger variance (**Figures S4B-C**). A critical exception was found at later stages of epidemic progression, when higher underestimations came from distributions with larger variance. This effect was driven by many exposed individuals taking longer to seroconvert than the mean time at a point in the epidemic when incidence events were becoming rarer. Compared to the results of **Figure S3**, AEU and REU were more sensitive to the mean rather than the shape of the T2S distribution.

| 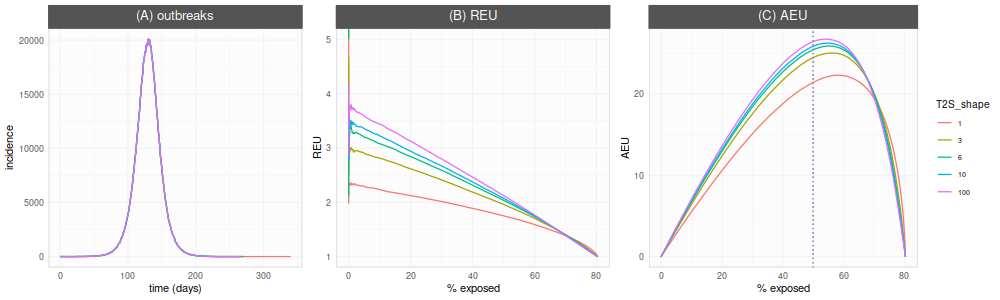  **Figure S4.** Same legend as Figure S1 but for varying $\Gamma s$. |
| --- |

Given that the rate at which incidence events are generated depend on the epidemic growth rate r, we looked at the sensitivity of REU and AEU to model parameters that affect r. We first varied R0, by keeping the infectious and incubation periods fixed (**Table 1**) and adjusting the transmission coefficient. Epidemic behaviour under different R0 values responded accordingly (**Figure S5A**). Both the AEU and REU presented a positive relationship with R0 (epidemic growth), with faster growing epidemics having the largest underestimations at any point of epidemic progression (**Figures S5B-C**).

| 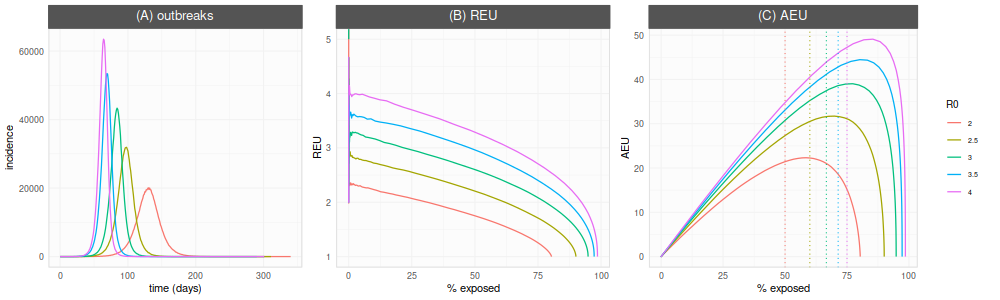  **Figure S5.** Same legend as Figure S1 but for varying R0. |
| --- |

Similar results were obtained when keeping R0 fixed but varying the infectious period (1/$\sigma$, note that in effect, fixing R0 and changing $\sigma$ results in an adjustment to the transmission coefficient $\beta$) (**Figure S6**). Shorter infectious periods (higher $\sigma$, thus higher $\beta$) resulted in faster developing epidemics (**Figure S6A**) with larger underestimations, while slower growing epidemics resulted in smaller underestimations (**Figures S6B-C**).

| 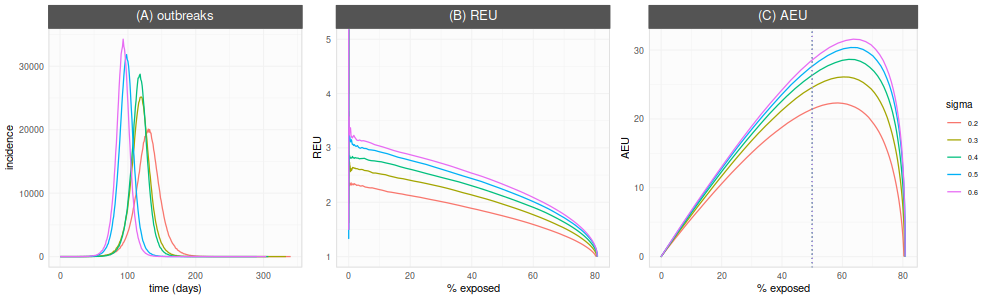  **Figure S6.** Same legend as Figure S1 but for varying $\sigma$. |
| --- |

The results in **Figures S4-6** were based on epidemic spread under an homogeneously mixing host-population. To assess the effects of meta-population structure on AEU and REU, the number of communities was varied from nC=1 to nC=900 (30^2 lattice). As expected from meta-population theory and the fact that only local transmission is modelled, increasing spatial structure had the general effect of slowing down epidemic growth and widening epidemic duration (**Figure S7A**). Following the results of **Figures S5-6** for R0 and infectious period, slower growing epidemics with more spatial structure had lower AEU and REU (**Figures S7B-C**).

| 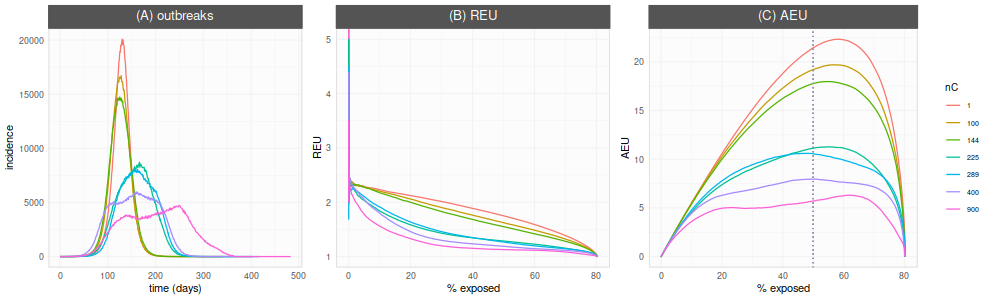  **Figure S7.** Same legend as Figure S1 but for varying nC. Note here that when increasing structure, stochasticity is important. These epidemics are single runs. |
| --- |

### **Comparison of model REU with predictive REU**

In the main text we show that pREU could explain ~94% of REU’s variation among the outbreaks generated with different host-pathogen characteristics (parameters). Here we present the effects of varying each parameter separately.

| 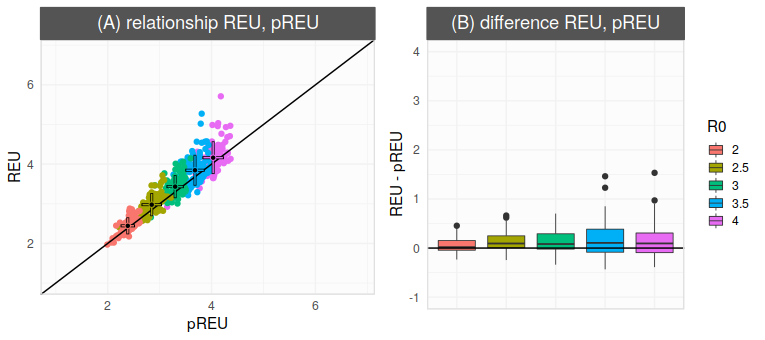 | **Figure S8.** REU and pREU for simulated outbreaks for which the R0 parameter was varied. (A) Relationship of REU and pREU. (B) Absolute difference between REU and pREU. |
| --- | --- |

| 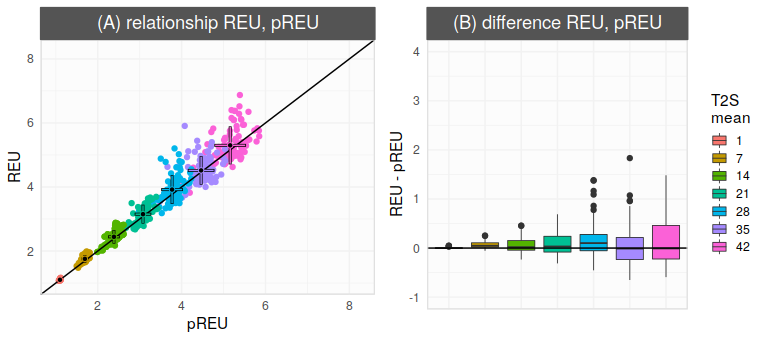 | **Figure S9.** REU and pREU for simulated outbreaks for which the T2S mean parameter was varied. (A) Relationship of REU and pREU. (B) Absolute difference between REU and pREU. |
| --- | --- |

| 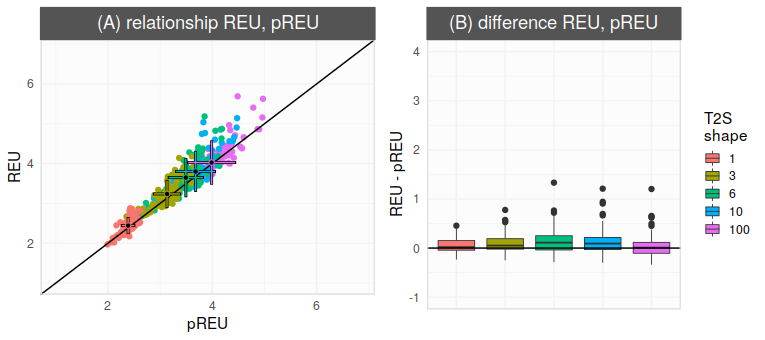 | **Figure S10.** REU and pREU for simulated outbreaks for which the T2S shape parameter was varied. (A) Relationship of REU and pREU. (B) Absolute difference between REU and pREU. |
| --- | --- |

| 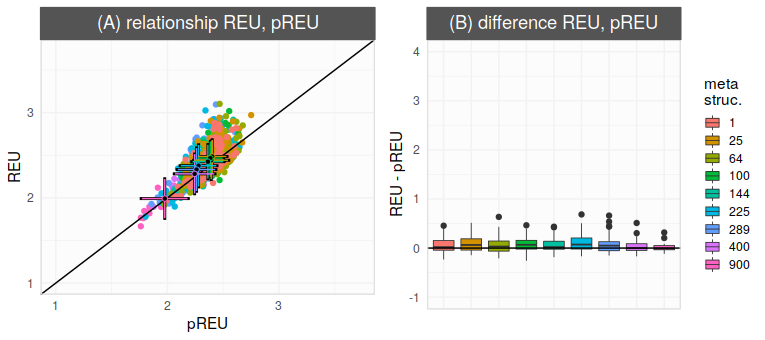 | **Figure S11.** REU and pREU for simulated outbreaks for which the nC parameter (population structure) was varied. (A) Relationship of REU and pREU. (B) Absolute difference between REU and pREU. |
| --- | --- |

### **Summarizing exposure underestimation during epidemic growth of second epidemic waves**

In the main text we show that pREU is able to predict the level of underestimation of exposure from seroprevalence during the growth phase of a first epidemic wave. However, for emerging pathogens, it is likely that other epidemic waves take place and that seroprevalence data is more readily available after the first wave. We thus looked at alternative scenarios to that of a single epidemic wave (**Figures 1-2**), by applying a control-related reduction in transmission for a fixed number of days starting during the first wave, effectively reducing its potential epidemic size while allowing for a second wave.

To allow for outbreak scenarios that develop into secondary epidemic waves, control of the first epidemic wave was modelled by implementing an instant reduction in transmission potential of 90% at tC (time of control start) for a duration of tCD (time of control duration): that is, we reduced the transmission coefficient $\beta=R_{0}(\sigma+1/LS)(\delta+1/LS)/\delta$ to $\beta'=0.1\beta$ (where LS is the average life-span). We modelled tC and tCD into two alternative scenarios; one in which the first wave was larger than the second, and another where the opposite took place. The starting day of control was different, but return to baseline transmission was set to the same day (respectively, for each scenario: tC=80 and tCD=50; tC=70 and tCD=60).

| 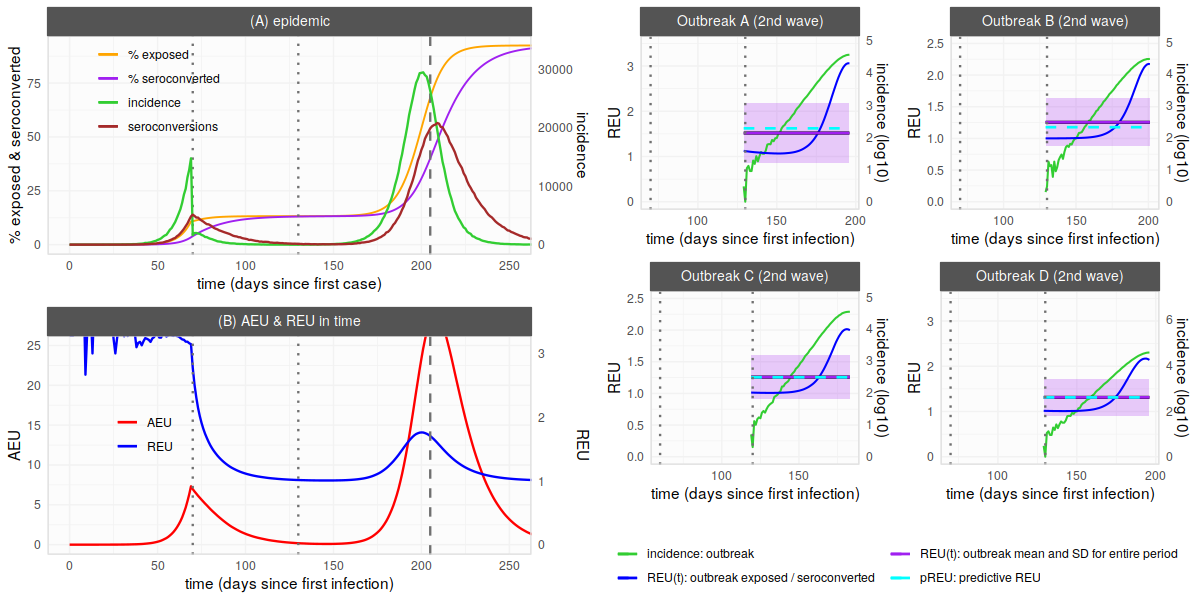 |
| --- |
| **Figure S12.** (A) Time series of cumulative incidence (exposed, orange), cumulative seroconverted (purple), incidence (green) and seroconversion (brown) daily events. (B) Relative exposure underestimation (REU, blue) and absolute exposure underestimation (AEU, red) dependent on time. Outbreak simulated with parameters as default in Table 1, when adding control. Control is modelled by caping transmission by 90% between days 70 and 130. (right) Examples of the first half of second waves from simulated outbreaks (A-D) with parameter variations from the default parameter set (Table 1). Outbreak A with $\Gamma m=28$. Outbreak B with $\Gamma s=6$. Outbreak C with $nC=100$. Outbreak D with $R_{0}=3$. All outbreaks with $R_{0}=3$. For each, the outbreak’s REU(t) (blue), incidence (green), REU mean and standard deviation for the entire time period (purple) and pREU (cyan) are presented. Time period considered is from the day control ceases to second wave peak. Control is modelled by caping transmission by 90% between days 70 and 130. |

###

| **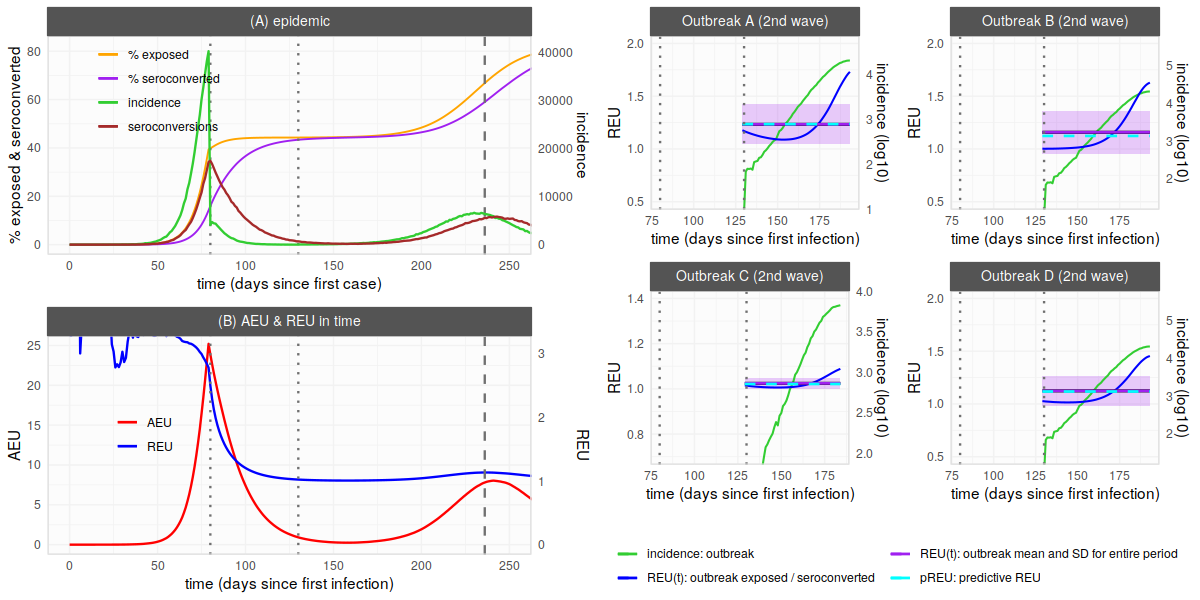** |
| --- |
| **Figure S13.** Same as Figure S10 but with control modelled by caping transmission by 90% between days 80 and 130. |

### **Sensitivity of pREU**

In the main text, Figure 3 summarises the sensitivity of pREU to its 3 parameters: growth rate r (ln(2)/DT), T2S mean and shape - in which the doubling times DT of 2, 4, 6 and 8 days are presented. Here, a wider range of doubling times is presented.

| **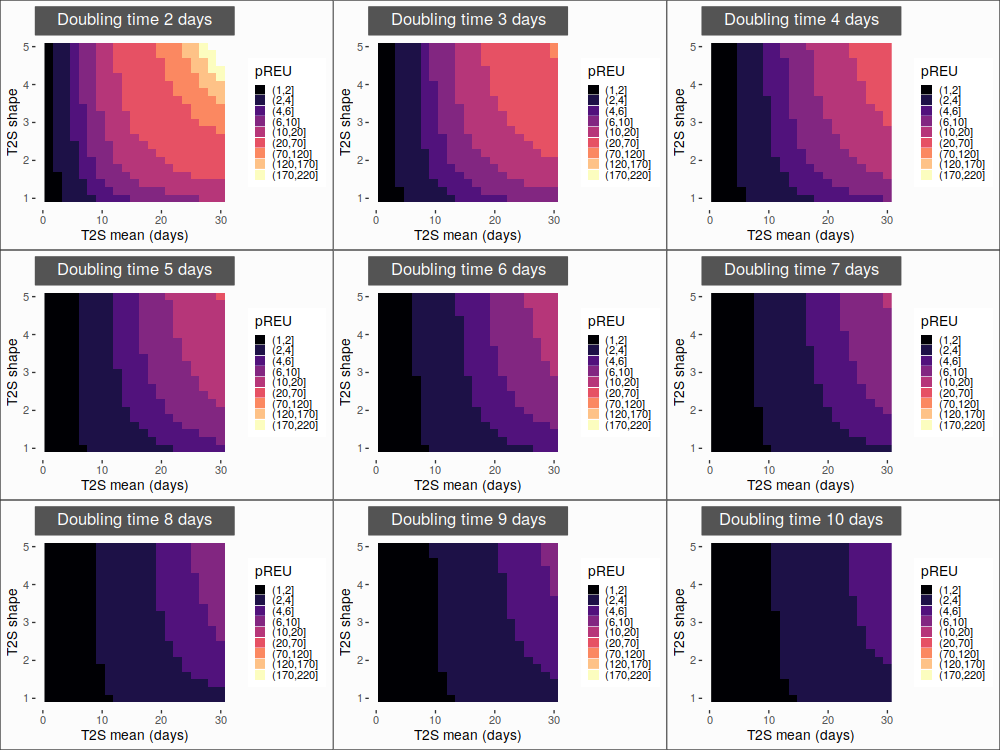** |
| --- |
| **Figure S14.** Sensitivity of pReu to T2S mean and shape, and doubling time (expression 4). pREU ranges are descritised in the color legend for visualization purposes. |
